# The association between affective temperaments and depressive symptoms in a population of medical university students, Poland

**DOI:** 10.3389/fpsyt.2023.1077940

**Published:** 2023-03-30

**Authors:** Natalia Karina Bartosik, Rafał Frankowski, Mateusz Kobierecki, Kacper Deska, Aleksander Twarowski, Bartłomiej Bąk, Marcin Kosmalski, Tadeusz Pietras

**Affiliations:** ^1^Students' Research Club, Department of Clinical Pharmacology, Medical University of Łódź, Łódź, Poland; ^2^Department of Clinical Pharmacology, Medical University of Łódź, Łódź, Poland

**Keywords:** depressive symptoms, depression, affective temperament, medical university students, mood disorder

## Abstract

**Background:**

Compared to their peers, medical students are more exposed to stress, and many present symptoms of depression, making them a group prone to experiencing mental illnesses.

**Objective:**

This study investigates a potential link between the occurrence of symptoms of depression and the dominating type of affective temperament in young people studying at a medical university.

**Methods:**

One hundred thirty-four medical students were surveyed using two validated questionnaires; the Polish versions of Beck’s Depression Inventory-II (BDI-II) and the Temperament Evaluation of the Memphis, Pisa, and San Diego Autoquestionnaire (TEMPS-A).

**Results:**

The data analysis revealed a significant link between symptoms of depression and affective temperaments, most significantly in subjects with an anxious temperament.

**Conclusion:**

This study confirms the role of various affective temperaments as a risk factor for mood disorders, specifically depression.

## 1. Introduction

Depression is a chronic (1) disorder affecting many people worldwide (2). Women are more susceptible to depression, suffering from this illness about twice as often as men (3). Age also affects the frequency of depression; the onset of the disease is most common in the second and third decades of life. This age range coincides with the studied subjects’ ages (3). In 2016 a 28% prevalence of depression in medical students was reported (4), alongside worse psychosocial well-being compared to undergraduate students in the normative sample (5). In a Mokros study assessing the prevalence of depression among medical students, 10% of participants obtained a clinically significant score of 17 or higher on the BDI (6). It is speculated that many medical students experience mental strain, which could predispose them to depression (7). Furthermore, a low number of medical students seek professional help due to the stigmatization associated with mental health disorders (8).

Early diagnosis and treatment of depression are imperative to protecting the individual’s quality of life and society’s functioning (9). The etiology of depression is multifactorial, including both environmental and genetic factors. A link has been found between the affective temperament (AT) type and the prevalence of depression (10). Affective temperaments are hypothesized to be a product of hereditary factors, hence the range of temperaments in different sexes and regions of the world (11). Interestingly, carriers of the s allele of the serotonin transporter gene score significantly higher in the TEMPS questionnaire, except in the hyperthymic temperament (12). It is widely accepted that the temperament of an individual forms in youth and remains fixed after that (13). Temperament may be a hereditary trait that makes individuals sensitized to negative stimuli throughout life (14). Research around affective temperaments presents an opportunity to screen people in risk groups for mood disorders (15); for example, certain temperament traits could indicate a predisposition or even subclinical depression (16). Potentially, temperaments may lay the foundation for affective disorders or constitute their subclinical form (17, 18). It is suggested that the habits and behaviors of an individual are the product of their temperament and environmental factors (19).

Akiskal split affective temperaments into five groups: depressive, hyperthymic, cyclothymic, irritable, and anxious. These increased the risk of affective disorders, including depression (17). On the other hand, there is speculation that a hyperthymic temperament can provide resistance to depression (20). The prognosis and course of depression could also be affected by one’s temperament (15, 21). A correlation exists between the type of temperament and the severity of one’s depressive symptoms (14). In conclusion, it is vital to investigate the relationship between the type of affective temperament and the rate of mood disorders/emotional problems in young people. Medical students are of particular interest due to their chronic stress exposure. This study aims to assess the link between affective temperaments and the prevalence of depression in a population of medical students. A study with more participants is needed before broader screening, and early intervention in depression can be introduced.

## 2. Methods

### 2.1. Participants

The participants recruited were students at the Medical University of Łódź, Poland. Participation in the study was voluntary; all students gave written, informed consent and did not receive compensation for their participation. The project obtained approval from the Ethics Committee at the Medical University of Łódź number RNN/260/21/KE.

Inclusion criteria were as follows: student status at the university; over 18. years of age; written and informed consent.

### 2.2. Measures

All participants provided primary socio-demographic data such as age, gender, year of study, and field of study. The severity of depressive symptoms was assessed using the BDI-II (22), a validated questionnaire with 21 items, each rated on a scale of 0–3 by the participant. According to a discriminant analysis concerning the Polish adaptation of Beck’s questionnaire, obtaining 0–16 points corresponds to no symptoms; 17–26 to moderate depression; 28–63 to severe depression (23); these ranges were used in the study.

The Polish version of the Temperament Scale of Memphis, Pisa, Paris, and San Diego-Autoquestionnaire (TEMPS-A) (24), translated by the Adult Psychiatry Clinic, Medical University of Poznań, was used. The translation was reviewed and accepted by Akiskal. It was used to assess affective temperaments, including depressive, cyclothymic, hyperthymic, irritable, and anxious, and contains 109 items for men and 110 for women (25).

### 2.3. Data collection

The data were collected from December 2021 to May 2022. An anonymous questionnaire was distributed to students after class by undergraduate researchers. Volunteers were invited to complete the study. Initial questions concerned the participants’ demographic traits, such as age; gender; field of study, and year of study. Participants then completed the BDI-II (22) and TEMPS-A (26). After collecting a sufficient number of correctly filled-out questionnaires, the data were analyzed.

### 2.4. Methods of data analysis

Scores from the BDI-II (22) and TEMPS-A (26) were used. Results were analyzed statistically using Statistica 13 software. Data was tested to assess completion of all individual statistical tests assumptions. The statistical method used was Chi-squared test to assess whether gender affects the prevalence of depression and whether medical students have a different prevalence of depression than other courses. The BDI-II score over 16 was marked as depressive symptoms and the lower score as absence of depressive symptomatology. Due to low levels of students with depressive symptomatology, Yates’s correction for continuity was used. Moreover, the two-tailed Mann–Whitney U test was also used in order to assess whether gender affects BDI-II score and whether medical students obtain a different BDI-II score than other courses. The value of p was set at <0.05. Spearman’s rank correlation test was used to identify the correlation between the levels of certain temperaments and BDI-II score. The correlation coefficient is denoted by ‘*r*.’

## 3. Results

Among 136 questionnaires, two were excluded due to a lack of written consent or incomplete responses. One hundred thirty-four questionnaires were subject to statistical analysis. In the sample: 82 participants (61.2%) were medical students, 25 (18.7%) physiotherapy students, and 27 (20.1%) students of other medical sciences. More detailed information on the study group is presented in Table 1. The participants were aged between 19 and 29. The average age of the assessed students was 22.43 years (SD = 1.7). Out of 134 participants, 15 scored above 16 points on the Beck Depression Scale, indicating clinically significant symptoms of depression. Four people had major depression, which is synonymous with obtaining above 26 points on the Beck Depression Scale. The other 119 participants failed to report any symptoms of depression on the questionnaire. The study revealed the presence of a depressive temperament in 18% of students; hyperthymic in 43.3%; cyclothymic in 13.4%; irritable in 3%, and anxious in 12.6%. 9.7% of participants scored the same number of points in more than one type of affective temperament. In males, a depressive temperament was present in 13.2% of individuals; hyperthymic in 51%; cyclothymic in 11.3%; irritable in 3.8%; and anxious in 9.4%. In the female group, a depressive temperament was present in 21.0% of participants; hyperthymic in 38.3%; cyclothymic in 14.8%; irritable in 2.5%; and anxious in 14.8%. 11.3% of males and 8.6% of females had more than one affective temperament. Affective temperament and depression severity are described in Table 2. The Mann–Whitney U test revealed that gender did not significantly affect the score obtained on the BDI-II (*p* = 0.15). Additionally, it did not affect the prevalence of depression (*p* = 0.75).

**Table 1:** Socio-demographic characteristics of the sample (*N* = 134).

Number of participants	134
Age (mean ± SD)	22.43 ± 1.7
Gender (*N*, %)	
Male	53 (39.6%)
Female	81 (60.4%)
Year of study (*N*, %)	
1	5 (3.7%)
2	26 (19.4%)
3	25 (18.7%)
4	52 (38.8%)
5	23 (17.2%)
6	3 (2.2%)
Field of study (*N*, %)	
Medicine	82 (61.2%)
Physiotherapy	25 (18.7%)
Electroradiology	13 (9.7%)
Medicine & dentistry faculty	7 (5.3%)
Emergency Medicine	4 (3%)
Public health	1 (0.7%)
Pharmacy	1 (0.7%)
Dental techniques	1 (0.7%)

SD, Standard deviation.

**Table 2:** Affective temperament and depression severity.

Affective temperament	No depression	Moderate depression	Severe depression
Cyclothymic (*N* = 18)	15 (83.3%)	2 (11.1%)	1 (5.6%)
Depressive (*N* = 24)	23 (95.8%)	0 (0%)	1 (4.2%)
Irritable (*N* = 4)	4 (100%)	0 (0%)	0 (0%)
Hyperthymic (*N* = 58)	56 (96.6%)	2 (3.4%)	0 (0%)
Anxious (*N* = 17)	11 (64.7%)	5 (29.4%)	1 (5.9%)
Mixed^*^ (*N* = 13)	10 (76.9%)	2 (15.4%)	1 (7.7%)

* There is more than one type of temperament.

The data showed that students from other courses scored statistically significantly higher on the BDI-II than medical students (*p* = 0.022). The average score obtained on the BDI-II by medical students was: 6.70, and for the other courses combined: 8.57.

The data analysis revealed a significant association between the BDI-II score (22) and particular types of affective temperament as measured by the TEMPS-A (26) within the student population of the medical university. A positive relationship between depressive symptoms with AT was revealed in a depressive temperament (*r* = 0.59, *p* < 0.001), cyclothymic temperament (*r* = 0.62, *p* < 0.001), irritable temperament (*r* = 0.51, *p* < 0.001); the most vital link was revealed with an anxious temperament (*r* = 0.68, *p* < 0.001). Depressive symptoms were weakly negatively associated with a hyperthymic temperament (*r* = −0.2, *p* = 0.02). The correlation between the levels of certain temperaments and depressive symptoms are described in Table 3.

**Table 3:** The correlation between the levels of certain temperaments and depressive symptoms.

Level of certain temperaments	*r*	*p* value
Depressive temperament	0.59	<0.001
Cyclothymic temperament	0.62	<0.001
Irritable temperament	0.51	<0.001
Anxious temperament	0.68	<0.001
Hyperthymic temperament	−0.2	0.02

*r* - Spearman’s rank correlation coefficient.

## 4. Discussion

To the best of our knowledge, this is one of the first studies to examine the relationship between various affective temperaments and the risk of experiencing depression in a population of medical students. The study revealed a link between the BDI-II score and the type of affective temperament, with the strongest association being observed in an anxious temperament. A positive correlation between depressive symptoms and temperament type was also revealed in depressive, cyclothymic, and irritable temperaments. A weak negative correlation was observed between higher scores obtained in the BDI-II and having a hyperthymic temperament. The results obtained correspond with the Baba et al. (27) study on 314 medical students and staff from hospitals and universities, which revealed a link between depression as measured by BDI-II score and some types of affective temperaments as measured by TEMPS-A. Temperaments associated with depressive symptoms include depressive, anxious, irritable, and cyclothymic temperaments. A hyperthymic temperament hada weak negative correlation (*r* = −0.2) with depressive symptoms in the tested sample (27). Our result concerning the interaction between a hyperthymic temperament and the prevalence of depressive symptoms is similar to the Lazary study results (28). Based on this information, further research in this area may be worthwhile.

Medical students are admitted to university based on their personality and intellect. High entry requirements and numerous factors negatively affecting mental well-being during medical school (including high competition, very high demands, and social pressure) may negatively affect the well-being of susceptible individuals (29). Some people will develop full depression as a result of these external factors. As the research has shown - the prevalence of depression in students sometimes reaches 25.6%, a significantly higher percentage than in data from the World Health Organization (30, 31). Our results are also in line with a study conducted by Kesebir et al. (10), which revealed that people suffering from depression received higher results in TEMPS-A in terms of depressive, cyclothymic, irritable, and anxious temperaments than people who are not proven to be depressed. Moreover, the Kesebir study showed that people diagnosed with depression score lower in the TEMPS-A regarding hyperthymic temperament (10). The above data are compatible with the Shahini study, which also revealed a negative correlation between a hyperthymic temperament and depressive symptoms and a positive correlation between depression and the previously mentioned temperament types (20). According to a study conducted on subjects from the general population in Hungary, the Zung Self-Rating Depression Scale (ZSDS) score was assessed with TEMPS-A results; anxious, depressive, and cyclothymic temperaments were in substantial positive correlation with depressive scores. Restrained interaction was also observed in irritable temperaments (28).

The process of developing depressive symptoms could be inhibited by a hyperthymic temperament, acting as a safeguard against the development of many psychiatric disorders, including depression (14). A study conducted on patients with major depressive disorder (MDD) who experienced no childhood trauma and were in remission during the study revealed a strong association between psychological resilience and hyperthymic temperament (32). Interestingly, research conducted by Gonda et al. revealed that exposure to recent stress has a similar effect as individual temperaments on developing depressive symptoms (33).

In a cross-sectional study conducted on a sample of elderly, non-psychiatric patients, a hyperthymic temperament was observed more often when compared to depressive patients.

Furthermore, the most substantial connection was observed between depression and a cyclothymic temperament, and the weakest between a hyperthymic temperament and depression (34).

Our study showed that males have a hyperthymic and irritable temperament more often than females. Conversely, females score higher in cyclothymic, anxious, and depressive temperaments. Our results align with the Borkowska et al. study on 521 Polish undergraduate students, which showed a sex difference in affective temperaments measured with the TEMPS-A. A more significant proportion of males than females possess a hyperthymic temperament. On the other hand, females score higher in anxious and depressive categories. These results are in line with similar studies from other countries, e.g., Yin et al., where: in the female group, there are more individuals with depressive, anxious, and cyclothymic temperaments, and in the male group, there are more individuals with hyperthymic and irritable temperaments (11, 35).

## 5. Limitations

The relatively small sample size increases the risk of selection bias and may affect the final result, therefore it may only reflect the tendencies of some medical students. Moreover, the medical students in Poland may be in a different mental state condition in comparison to students from other countries, which may not reflect the general tendency around the world. The results are also not reflective of the relationship between AT and depressive symptoms at other medical schools, as the study was only carried out at the Medical University of Łódź. The participants were students of medical sciences only, so patterns among students of other courses remain unknown. Our research was carried out from December 2021 until May 2022, which may have an impact on the results. Many mental stressors identified could have impacted the comfort and mental health of the participants at this time (7). As numerous studies have shown: the COVID-19 pandemic affects the onset of depression and anxiety in the population (30). In addition, medical students are burdened with a greater load - the stress associated with remote teaching and isolation, the prospect of working with sick patients and the fear of transmitting the virus to relatives may worsen their medical condition (31). This time also coincides with term exams and the outbreak of a war in Ukraine, a period of increased stress for students, which may impact the final results. Moreover, it is the period of the highest intensity of depressive symptoms in the course of seasonal affective disorder (36). Additionally, a significant limitation of the study was no inquiry into the current or past psychiatric treatment of the participants, which may have affected the BDI-II (22) and TEMPS-A scores (26). Additionally, the assessment of affective temperament with the TEMPS-A may have been affected by the participants’ frame of mind (37). Potentially, participants with cyclothymic or low anxious temperaments may also suffer from hypomanic or manic episodes (38). Recurrent and early onset major depressive episodes are both predictors of bipolarity (39); therefore, it is impossible to state if some participants suffer from depression or bipolar disorder. This study lacks information regarding lifetime depression or hypomania.

## 6. Conclusion

Our data reveal a positive correlation between every affective temperament, except hyperthermic, and the severity of depressive symptoms among students. A hyperthymic temperament may constitute protection against the onset of depressive symptoms among medical students. A study examining a larger sample size with fewer social stressors and engaging medical students also around the world is needed. Participants with prior and current psychiatric interventions should also be accounted for or excluded from future studies.

## Data availability statement

The raw data supporting the conclusions of this article will be made available by the authors, without undue reservation.

## Ethics statement

The research obtained approval from the Ethics Committee of the Medical University of Lodz number RNN/260/21/KE. All students provided written informed consent to participate.

## Author contributions

NB and RF: literature collection, literature review, and manuscript preparation. BB and KD: manuscript preparation. AT: language consultation and manuscript preparation. MK: statistical consultation and research supervision. TP: study design and research supervision. All authors contributed to the article and approved the submitted version.

## Funding

This research was funded by the Medical University of Lodz, institutional grant no. 503/1-151-07/503-11-001-18.

## Conflict of interest

The authors declare that the research was conducted in the absence of any commercial or financial relationships that could be construed as a potential conflict of interest.

## Publisher’s note

All claims expressed in this article are solely those of the authors and do not necessarily represent those of their affiliated organizations, or those of the publisher, the editors and the reviewers. Any product that may be evaluated in this article, or claim that may be made by its manufacturer, is not guaranteed or endorsed by the publisher.
